# Frequency and clinical characteristics of children and young people with type 2 diabetes at diagnosis from five world regions between 2012 and 2021: data from the SWEET Registry

**DOI:** 10.1007/s00125-024-06283-5

**Published:** 2024-11-06

**Authors:** Rosaria Gesuita, Alexander J. Eckert, Stéphane Besançon, Nancy A. Crimmins, Fred Cavallo, Jaehyun Kim, Craig Jefferies, Evelien F. Gevers, Anastasios Vamvakis, Sejal Shah, Shazhan Amed, Valentino Cherubini, on behalf of the SWEET study group

**Affiliations:** 1 Center of Epidemiology, Biostatistics and Medical Information Technology, Università Politecnica delle Marche, Ancona, Italy; 2 IRCCS INRCA, Ancona, Italy; 3 Institute of Epidemiology and Medical Biometry, Ulm University, Ulm, Germany; 4 German Centre for Diabetes Research (DZD), Neuherberg, Germany; 5 NGO Santé Diabète, Bamako, Mali and Unité PACRI, Conservatoire National des Arts et Métiers, Paris, France; 6 Division of Pediatric Endocrinology, Cincinnati Children’s Hospital Medical Center, Cincinnati, OH USA; 7 University of Cincinnati School of Medicine, Cincinnati, OH USA; 8 Endocrinology Service, National Children’s Hospital, San José, Costa Rica; 9 Department of Pediatrics, Seoul National University Bundang Hospital, Seongnam, Korea; 10 Te Whatu Ora, Health NZ, Starship Children’s Health, Auckland, New Zealand; 11 Centre for Endocrinology, William Harvey Research Instititute, Queen Mary University of London, London, UK; 12 Department of Paediatric Endocrinology and Diabetes, Royal London Children’s Hospital, Barts Health NHS Trust, London, UK; 13 Pediatric Endocrine Unit, 3rd Pediatric Department, Aristotle University, Hippokration General Hospital, Thessaloniki, Greece; 14 Division of Pediatric Endocrinology, Stanford University, Stanford, CA USA; 15 Division of Endocrinology, Department of Pediatrics, BC Children’s Hospital, Vancouver, BC Canada; 16 Department of Women’s and Children’s Health, Azienda Ospedaliero/Universitaria delle Marche, ‘G. Salesi Hospital’, Ancona, Italy

**Keywords:** Adolescents, Children, Geographical variability, Temporal trends, Type 2 diabetes

## Abstract

**Aims/hypothesis:**

The diagnosis of type 2 diabetes is increasing in young people worldwide. This study evaluated the frequency and clinical characteristics of young people presenting with type 2 diabetes from the multinational SWEET e.V Registry 2012–2021, including the first years of the COVID-19 pandemic.

**Methods:**

This is a longitudinal observational study based on the SWEET Registry, which collects demographic and clinical data on children and adolescents with diabetes from centres worldwide, with the diagnosis and classification of diabetes provided locally by each centre according to International Society for Paediatric and Adolescent Diabetes definitions. By July 2022, the SWEET Registry included 96,931 individuals from 130 centres with a total of 1,154,555 visits. Data were analysed by region: Europe (EU), Australia and New Zealand (AU/NZ), South America (SA), North America (NA) and Asia/Middle East and Africa (AS/AF). Trends in proportions for the two-year periods, calculated as cases with type 2 diabetes diagnoses over all cases with diabetes diagnoses, were estimated using logistic regression models adjusted for age at onset and sex.

**Results:**

Overall, there were 2819 of 58,170 new cases (4.8%) with type 2 diabetes: 614 in EU, 293 in AU/NZ, 79 in SA, 1211 in NA and 622 in AS/AF. The proportion of type 2 diabetes increased from 3.2% to 6.0% from 2012/2013 to 2020/2021, a relative rate of increase of 9% per two-year period (95% CI 5.9, 12.3; *p*<0.001). In the two-year period of the COVID-19 pandemic, type 2 diabetes continued to follow the observed trend, with a proportion of 6.0% in 2020–2021 compared with 5.4% in 2018–2019. High variability in the proportion of type 2 diabetes was observed across regions, with the lowest values observed in EU and the highest in NA. A significant increase in the proportion of type 2 diabetes was observed in EU, AU/NZ and NA. The median HbA_1c_ was not uniform and was highest in AS/AF (85 mmol/mol [9.9%]; IQR 55–111 [7.2–12.3%]) and lowest in EU (63 mmol/mol [7.9%]; IQR 48–99 [6.5–11.2%]), and the difference between EU and NA (median value 73 mmol/mol [8.8%]; IQR 50–105 [6.7–11.8%]) was statistically significant (*p*=0.047). There was also a difference in BMI SD score by region: the lowest median BMI SD score was 2.2 (IQR 1.4–2.7) in AS/AF and the highest was 3.1 (IQR 2.5–3.6) in AU/NZ.

**Conclusions/interpretation:**

The multinational SWEET data from the years 2012 to 2021 inclusive support recent findings of a worldwide increase in type 2 diabetes in young people, albeit with regional differences. This increase highlights the need for ongoing preventive measures and available advanced treatment modalities worldwide.

**Graphical Abstract:**

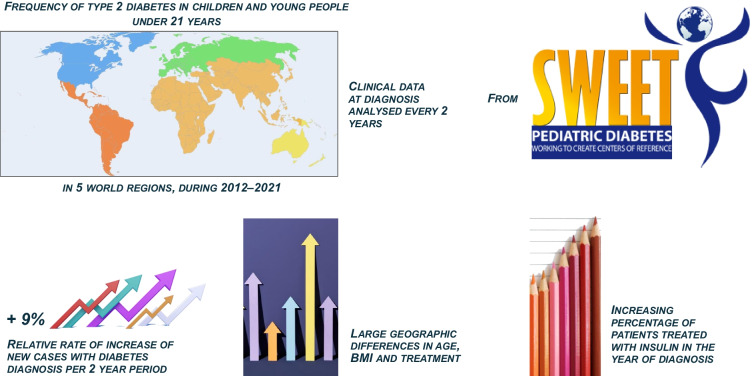

**Supplementary Information:**

The online version of this article (10.1007/s00125-024-06283-5) contains peer-reviewed but unedited supplementary material.


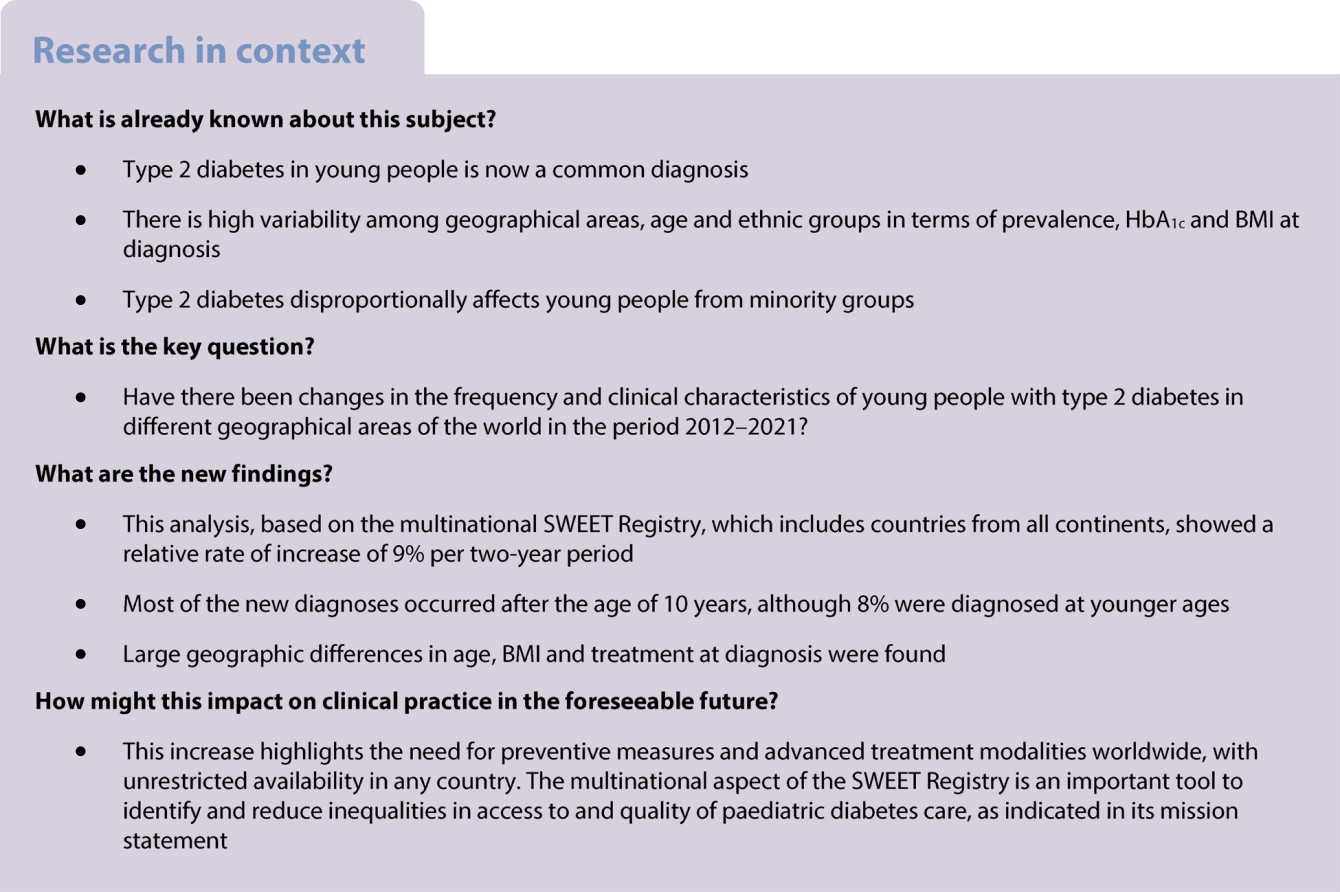


## Introduction

Type 2 diabetes in young people is increasing, with both the incidence and prevalence increasing over the last few decades in many countries; however, there is high variability according to geographical area, age and ethnic group [1–4]. The increase in young people with type 2 diabetes is partly concurrent with the increasing obesity in young people observed in many countries [2]. Type 2 diabetes with onset in childhood or adolescence is more aggressive than adult-onset type 2 diabetes, with a higher risk of long-term complications and comorbidities, as well as premature mortality compared with youth-onset type 1 diabetes [3, 5] and reduced overall health and quality of life. Therefore, type 2 diabetes in young people is a clinical, societal and economic burden, with a high impact on health systems. Analysis of the clinical characteristics at diagnosis for patients with type 2 diabetes is of great importance to identify possible prevention strategies and the most effective treatments.

The SWEET e.V. Registry (Better Control in Paediatric and Adolescent Diabete**S**: **W**orking to cr**E**ate C**E**n**T**ers of Reference), launched in 2008 with the aim of harmonising the care of children with all types of diabetes in Europe, has expanded worldwide since 2011, and included 130 centres in 2022. Participating centres prospectively collect quality data that are used to produce standardised benchmarking reports. Non-corporate members of the SWEET Registry are listed in the electronic supplementary material (ESM).

We aimed to evaluate the frequency and clinical characteristics of young people with type 2 diabetes at diagnosis using data from the SWEET Registry over a 10-year period.

## Methods

### Data sources and participants

Data were obtained from the international, multicentre SWEET Registry, a worldwide network of centres for the care of people living with diabetes under 25 years old. It was established as a European project in 2008, and in 2011 became a non-profit worldwide initiative supported by the International Society for Paediatric and Adolescent Diabetes (ISPAD).

The SWEET Registry collects demographic and clinical data from participating centres around the world. Diagnosis of diabetes and classification of diabetes types are provided locally by each centre according to ISPAD definitions [6]. Data are collected locally through clinical databases, and then aggregated into an anonymised cumulative database that is used for clinical research, scientific analysis and international benchmarking. Twice a year, each participating centre exports standardised data to the Institute of Epidemiology and Medical Biometry (Ulm University, Germany), in accordance with data protection regulations and ethics requirements. The Institute of Epidemiology and Medical Biometry is also responsible for statistical analysis of the SWEET data and for the SWEET-DPV software that is used by some participating SWEET centres for diabetes-specific medical documentation. Verbal or written informed consent was obtained from individuals or their guardians based on national regulations in the respective countries. The study was carried out in accordance with the Declaration of Helsinki.

We extracted all newly diagnosed diabetes cases of any type in individuals who were diagnosed under 21 years old from the SWEET Registry, with a diagnosis date between 2012 and 2021 inclusive.

The inclusion criteria were individuals who received a new diagnosis of diabetes between 2012 and 2021, and were under 21 years old at the time of diagnosis. The exclusion criteria included a diagnosis of pre-diabetes.

### Variables

BMI was analysed as an SD score (BMI-SDS), calculated using the WHO BMI charts [7]. Metabolic control was assessed on the basis of HbA_1c_, which was measured locally in each centre. To adjust for differences between laboratories, the multiple of the mean method was used to mathematically standardise HbA_1c_ values to the reference range of the DCCT (21–43 mmol/mol [4–6%]) [8].

To stratify the analysis for the various regions, we grouped participants based on the country of the centre they were treated in. We defined five regions, namely Europe (EU), Australia and New Zealand (AU/NZ), South America (SA), North America (NA) and Asia/Middle East and Africa (AS/AF).

### Statistical analysis

Unadjusted comparisons between the two-year intervals and between the five regions were performed for data at diagnosis (using aggregated data for each patient within the respective year of diagnosis). Results are presented as median (IQR) or as percentages. *p* values were generated using the Wilcoxon rank sum test for quantitative variables and the χ^2^ test for qualitative variables. The Bonferroni–Holm method was used to adjust for multiple comparisons. The *p* value significance threshold was set at *p*<0.05.

To investigate the trend in the proportion of newly diagnosed type 2 diabetes among all patients diagnosed with any diabetes type, data were analysed in two-year increments, as the number of participants in some regions was too low for annual analyses. Trends in proportions for the two-year periods were calculated using logistic regression models adjusted for sex and age at onset (grouped as <6, 6 to <12 and 12 to <21 years).

To estimate the associations of HbA_1c_ at diagnosis and BMI-SDS within the first year after diagnosis with sex or age at diagnosis (<12, 12–14, 15–20 years) among the patients with newly diagnosed type 2 diabetes, linear regression models (with random intercept) were used, adjusted by age group or sex. The same approach was used when analysing the proportion of patients receiving injectable/oral glucose-lowering agents or insulin treatment within the first year after diagnosis using logistic regression models. To calculate the annual increase in mean HbA_1c_ at diagnosis among the patients with newly diagnosed type 2 diabetes, a linear trend regression model was used. This regression model was adjusted for age at onset and sex, and a random intercept was implemented to take into account centre heterogeneity.

## Results

### At diagnosis

**Fig. 1:**
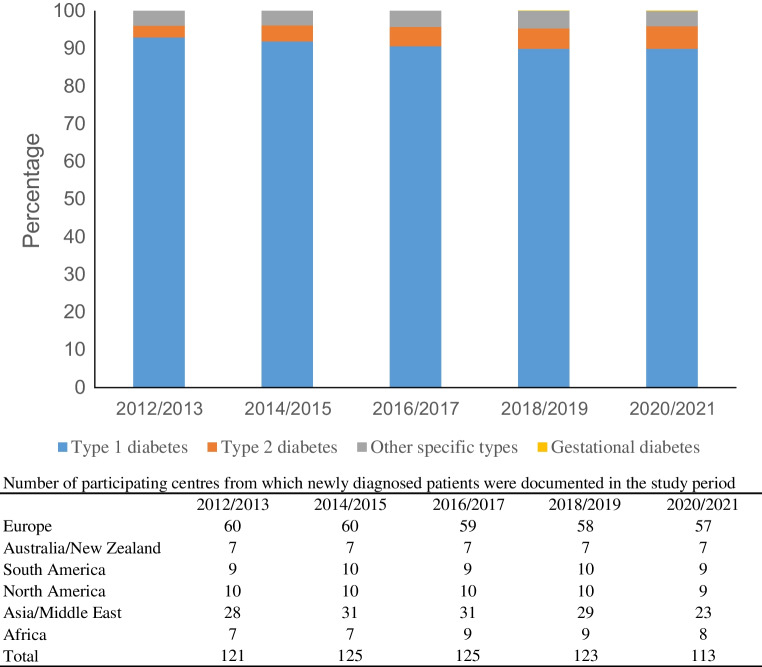
Distribution according to type of diabetes

The SWEET Registry included 96,931 patients from 130 centres, with 1,154,555 visits in the study time frame (2012 to 2021). Of the 58,170 new cases with diabetes, 2819 had type 2 diabetes (4.8%). The proportion of newly diagnosed type 2 diabetes registered in the SWEET database increased from 3.2% to 6.0% from 2012/2013 to 2020/2021, representing a relative rate of increase of 9% per two-year period (95% CI 5.9, 12.3; *p*<0.001). Table 1 shows the number of children and adolescents with type 2 diabetes by year of onset and geographical area. Figure 1 shows the distribution of children and adolescents according to diabetes type and the number of participating centres in the study by geographical area over the study period.

**Table 1:** Number and proportion of children and adolescents with newly diagnosed type 2 diabetes by year of onset and region

	2012/2013	2014/2015	2016/2017	2018/2019	2020/21	Total
EU	73 (1.4)	92 (1.6)	141 (2.2)	157 (2.6)	151 (2.6)	614
AU/NZ	32 (5.8)	52 (7.9)	58 (8.6)	70 (9.5)	81 (12.2)	293
SA	8 (2.9)	19 (5.3)	13 (3.2)	19 (5.8)	20 (6.3)	79
NA	157 (7.0)	211 (8.2)	274 (9.6)	206 (7.7)	363 (13.2)	1211
AS/AF	47 (2.9)	120 (5.5)	181 (7.0)	202 (8.5)	72 (4.1)	622
Total	317	494	667	654	687	2819

Values are *n* (%)

Percentages are calculated by dividing the number of new diagnoses of type 2 diabetes by year of onset and region by the number of all new cases of diabetes by year of onset and region

Over the same period, the proportion of type 1 diabetes cases among all patients newly diagnosed with any diabetes type, documented in the SWEET Registry, decreased from approximately 93% to 90%, while the proportion of other specific types of diabetes did not change significantly over time.

**Fig. 2:**
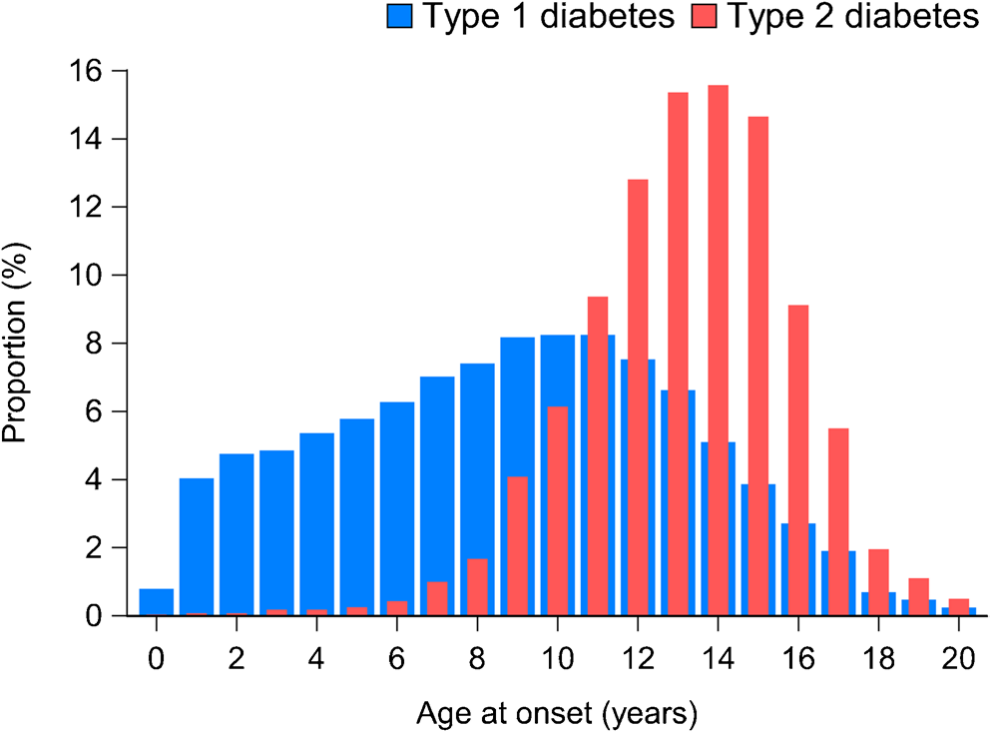
Distribution of patients with type 1 and type 2 diabetes by age at diagnosis in the period 2012–2021, showing the proportion (%) of patients diagnosed at each age

Figure 2 shows the distribution of type 1 and type 2 diabetes by age at diagnosis over the whole period (2012–2021). The distribution by age was quite different between those with type 1 diabetes and those with type 2 diabetes. Of all children with type 2 diabetes, 224 (8%) were <10 years old at diagnosis; as expected, the proportion progressively increased with age, with a peak between 13 and 15 years old.

In the two-year period of the COVID-19 pandemic, the proportion of patients with type 2 diabetes appeared to follow the trend observed between 2012 and 2019, with a proportion of 6.0% in 2020–2021 compared with 5.4% in 2018–2019, therefore demonstrating a similar increase as in the previous two-year periods. During the COVID period, 2020–2021, an increase in the proportion of newly type 2 diabetes diagnosis among all new patients with diabetes documented in the SWEET Registry was observed in AU/NZ and NA.

Table 2 shows the mean and 95% CI for HbA_1c_ at diagnosis and the BMI-SDS within the first year after diagnosis by sex and age at diagnosis. A significant difference was found between sexes for mean HbA_1c_, with male participants having a higher mean than female participants, while no significant difference was found between ages at diagnosis. Significant differences were found in BMI-SDS, with male participants and those in the younger age group having higher mean values than female participants and those in the older age groups, respectively.

**Table 2:** HbA_1c_ and BMI-SDS at diagnosis by sex and age at year of diagnosis

	HbA_1c_ (mmol/mol)		BMI-SDS	
		*p* value		*p* value
*N*	829		1570	
Sex^a^				
Female	77.8 (71.7, 83.9)	0.016	2.5 (2.3, 2.7)	0.001
Male	83.4 (77.1, 89.8)		2.7 (2.5, 2.8)	
Age at diagnosis (years)^b^				
<12	78.4 (71.2, 85.5)	0.592	2.8 (2.6, 3.0)	<0.001
12–14	80.0 (73.7, 86.3)		2.5 (2.4, 2.7)	
15–20	81.5 (75.1, 87.9)		2.5 (2.3, 2.7)	

Values are mean (95% CI)

Results were obtained using linear regression analysis

^a^Adjusted for age at diagnosis

^b^Adjusted for sex

A total of 1746 children and adolescents with type 2 diabetes were treated within the first year after diagnosis, including 958 (55%) who were treated with oral glucose-lowering drugs, 25 (1%) who were treated with injectable glucose-lowering drugs, 969 (55%) who were treated with either oral or injectable glucose-lowering drugs or both, 512 (29%) who were treated with insulin, and 223 (13%) who were treated simultaneously with glucose-lowering drugs (oral or injectable) and insulin. The median time from diagnosis to use of the first glucose-lowering drug was 25 days (IQR 0–67) and median time from diagnosis to the first use of insulin was 22 days (IQR 0–66). The time from first use of a glucose-lowering drug to first use of insulin, in those who did not start them simultaneously, was 52 days (IQR 31–95). Table 3 shows the proportion of injectable/oral glucose-lowering drugs and insulin by sex and age at diagnosis within the first year after diagnosis. No significant differences were found between sexes or age groups.

**Table 3:** Proportions of children receiving injectable/oral glucose-lowering agents and insulin treatment within the first year after diagnosis by sex and age at year of diagnosis

	Injectable/oral glucose-lowering drugs	*p* value	Insulin	*p* value
*n*	1746		1746	
Sex^a^				
Female	49.5 (42.2, 56.8)	0.106	34.0 (27.8, 40.7)	0.082
Male	53.5 (45.9, 60.9)		38.2 (31.4, 45.4)	
Age at diagnosis (years)^b^				
<12	48.5 (40.1, 57.0)	0.514	33.9 (26.7, 41.9)	0.122
12–14	52.1 (44.6, 59.5)		38.7 (32.0, 45.9)	
15–20	51.9 (44.3, 59.5)		33.7 (27.3, 40.1)	

Values are percentages (95% CI)

Results were obtained using logistic regression analysis

^a^Adjusted for age at diagnosis

^b^Adjusted for sex

### Regional distribution by year of diagnosis

**Fig. 3:**
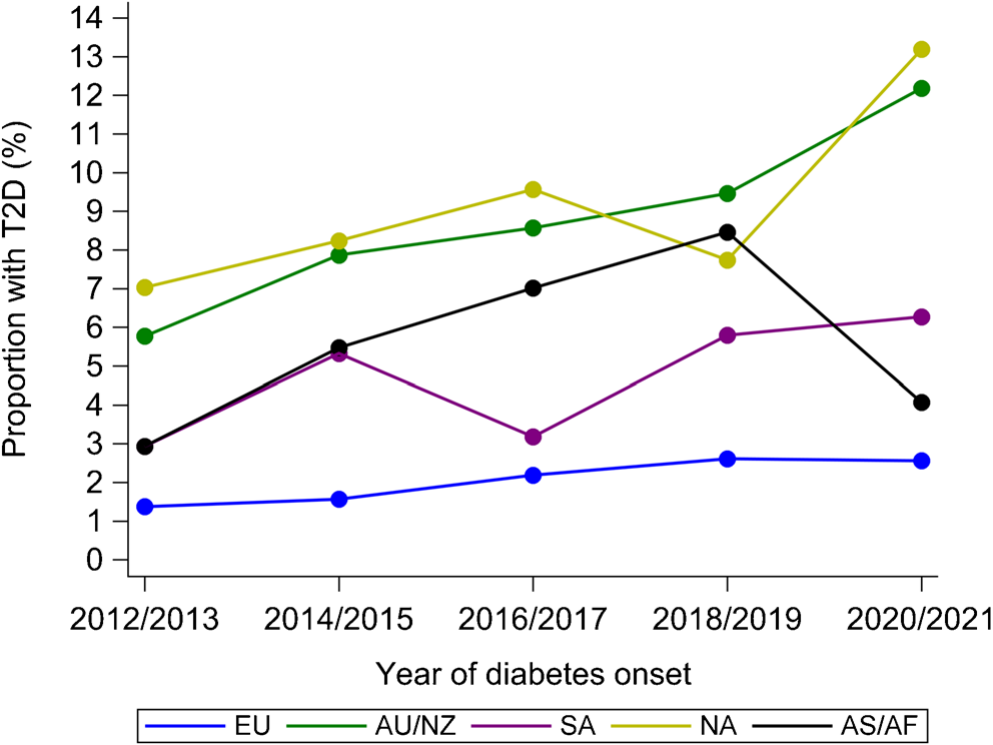
Percentage of new patients with type 2 diabetes (T2D) among all diabetes cases by region and period of diagnosis

The proportion of type 2 diabetes significantly increased in the study period (Table 4), with a relative percentage change of 9.0% overall (95% CI 5.9, 12.3), and a higher increase in male participants than female participants, and in adolescents aged 15–20 years than in other age groups. A significant increase in the proportion of type 2 diabetes was observed in EU, AU/NZ and NA. The trend observed in AS/AF over the entire period was not statistically significant, with an increase from 2012 to 2019 and a decrease in the 2020–2021 period. Limiting the trend analysis to the period 2012–2019, a significant increase of 29.7% (95% CI 18.6, 41.8) was observed in AS/AF. Figure 3 shows the trend in the frequency of type 2 diabetes by region. A high variability in the proportion of adolescents with type 2 diabetes was observed across the regions in the study period, with the lowest values observed in EU.

**Table 4:** Trends in proportion of patients with type 2 diabetes (%) by sex, age at diagnosis and region

	Relative percentage change^a^	95% CI	*p* value
Overall^b^	9.0	5.9, 12.3	<0.001
Sex^c^			
Male	16.1	10.9, 21.5	<0.001
Female	4.0	0.02, 8.1	0.049
Age at diagnosis (years)^d^			
<12	−23.4	−44.3, 5.6	0.102
12–14	5.4	−0.5, 11.6	0.075
15–20	10.8	7.0, 14.7	<0.001
Region^b^			
EU	10.4	3.8, 17.3	0.002
AU/NZ	13.6	3.5, 24.7	0.007
SA	13.9	0.95, 35.8	0.145
NA	8.7	3.8, 14.0	<0.001
AS/AF	5.9	0.99, 13.1	0.085

Results were obtained using linear regression analysis

^a^Per two-year interval from 2012–2013 to 2020–2021

^b^Adjusted for age at diagnosis and sex

^c^Adjusted for age at diagnosis

^d^Adjusted for sex

### Patient characteristics at diagnosis in two-year steps

**Fig. 4:**
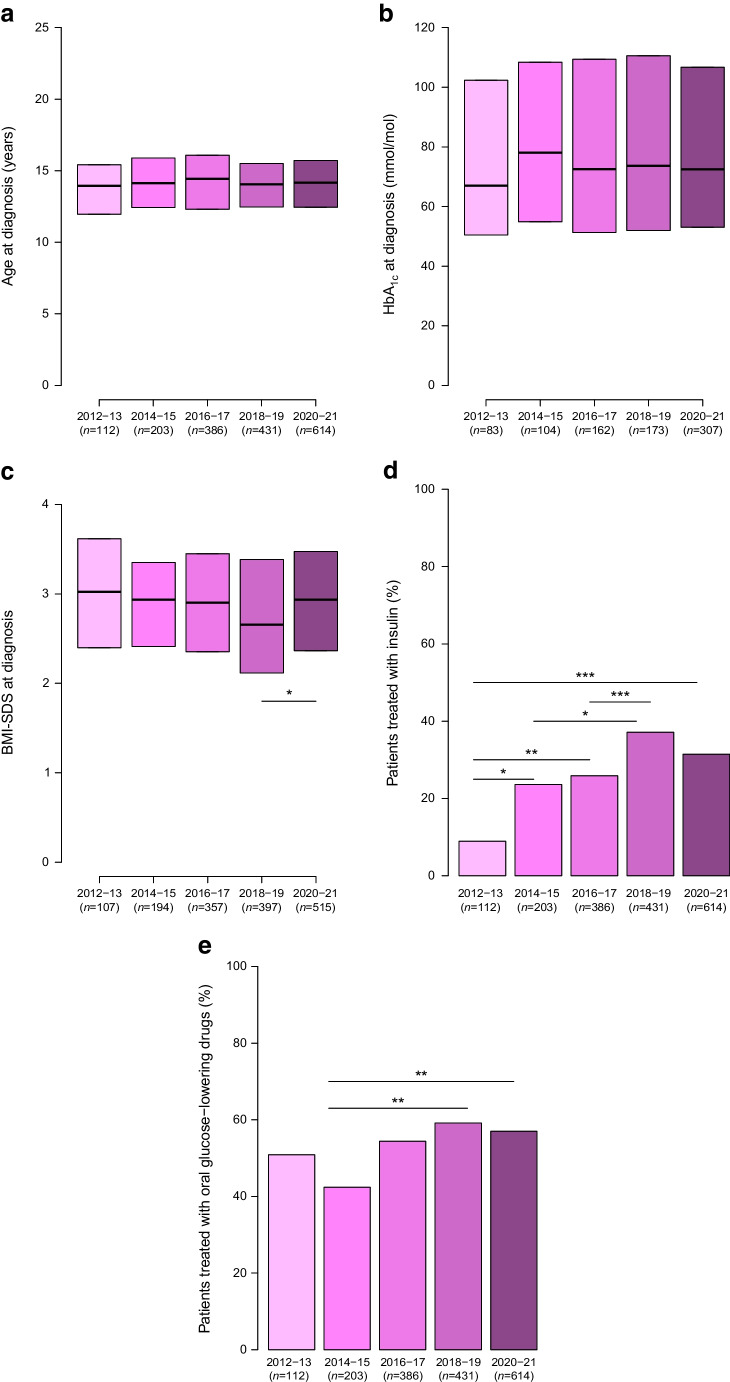
Key clinical characteristics of patients at diagnosis by period of diagnosis. Asterisks indicate statistically significant differences between periods: *p<0.05, **p<0.01, ***p<0.001. Data are medians and IQR in (**a**–**c**) and proportions in (**d**) and (**e**)

Figure 4 shows the key patient characteristics for each two-year period of diagnosis. The median HbA_1c_ ranged between 67 and 78 mmol/mol (8.3–9.3%) (Fig. 4b), with a non-significant age- and sex-adjusted annual increase of 0.58 mmol/mol (95% CI −0.28, 1.45). The median BMI-SDS (Fig. 4c) was fairly stable over the study period at the value of approximately 3. The proportion of patients with a diagnosis of type 2 diabetes who were treated with insulin (Fig. 4d) increased from 8.9% in 2012–2013 to 37% in 2018–2019 and 31% in 2020–2021, and the proportion treated with oral agents (Fig. 4e) increased from 51% in 2012–2013 to 57% in 2020–2021.

### Patient characteristics at diagnosis by region

**Fig. 5:**
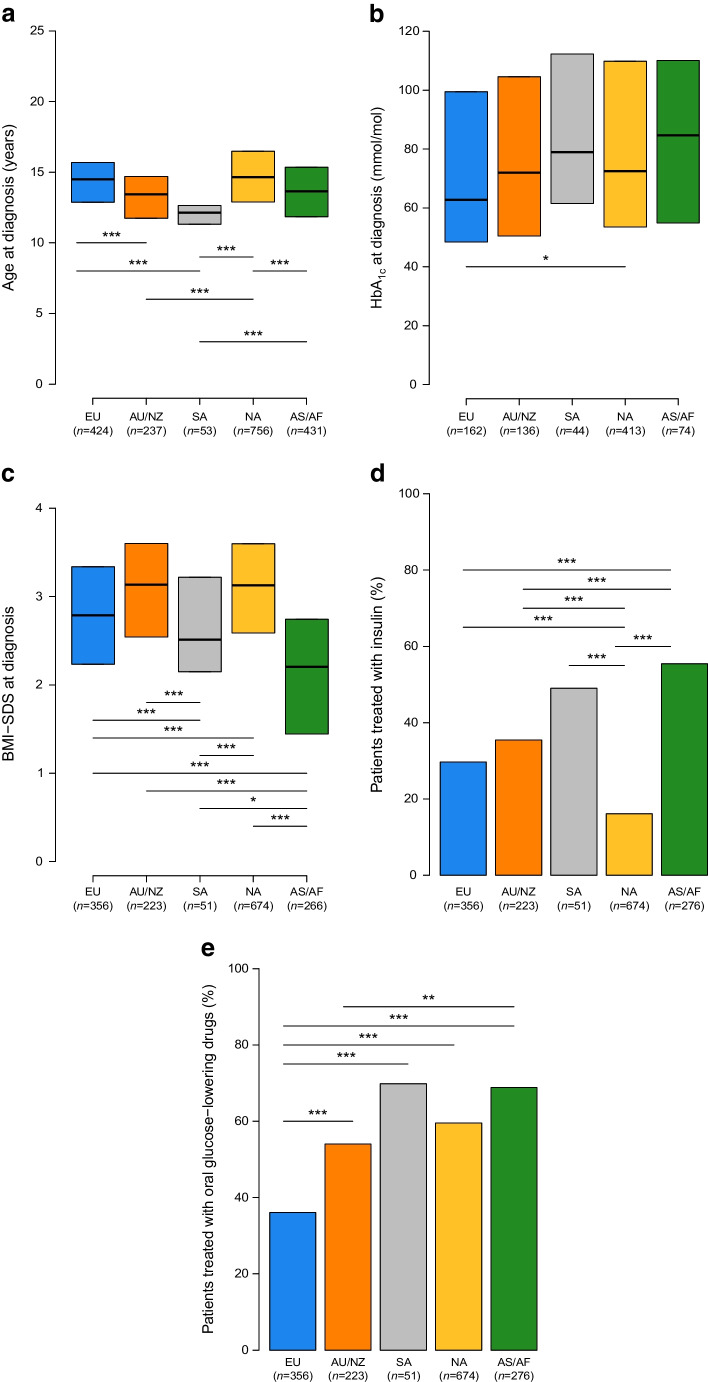
Key clinical characteristics of patients at diagnosis by regions. Asterisks indicate statistically significant differences between regions: *p<0.05, **p<0.01, ***p<0.001. Data are medians and IQR in (**a**–**c**) and proportions in (**d**) and (**e**)

Figure 5 shows the key patient characteristics at diagnosis by region. The median age at diagnosis (Fig. 5a) ranged between 12.1 years in SA and 14.7 years in NA, with significant differences among regions, except between EU and NA and between AU/NZ and AS/AF.

The highest median HbA_1c_ (Fig. 5b) was 85 mmol/mol (9.9%) in AS/AF and the lowest was 63 mmol/mol (7.9%) in EU; a significant difference in HbA_1c_ values was found between EU and NA (for which the median value was 73 mmol/mol [8.8%]) (*p*=0.047). The lowest median BMI-SDS (Fig. 5c) was 2.20 in AS/AF and the highest was 3.14 in AU/NZ, with significant differences among regions, except between EU and SA and between AU/NZ and NA.

Use of diabetes medication at onset of type 2 diabetes also showed regional variability, with the percentage of patients treated with oral glucose-lowering drugs (Fig. 5e) ranging from 36.1% in EU to 69.8% in SA, while the percentage of patients treated with insulin (Fig. 5d) ranged from 16.1% in NA to 55.4% in AS/AF. The proportion of patients treated with other injectable hyperglycaemic agents, such as glucagon-like peptide 1 GLP-1 receptor agonists, in the study period was low at 1.4% in EU and 2.5% in NA, and zero in the other world regions. The overall proportion patients treated with these agents was 0% in 2012/2013, and increased to 2.1% in 2020/2021.

Because the AF/AS area includes at least three ethnic groups (Asian, African and people from the Middle East), we compared data on BMI-SDS, HbA_1c_ and treatment between patients in Africa (*n*=23), Asia (*n*=208) and the Middle East (*n*=45). No significant differences were found between the three regions in terms of BMI-SDS (Asia: 2.19, IQR 1.45–2.71; Middle East 2.40, IQR 1.53–3.11; Africa 2.19, IQR 1.10–2.97; *p*=0.999), but Asian patients had a significantly higher median HbA_1c_ than Middle Eastern patients (Asia: 100.7 [11.4%], IQR 82.5–116.7 [9.7–12.8%]; Middle East: 66.3 [8.2%], IQR 48.7–91.5 [6.6–10.5%]; Africa 106.7 [11.9%], IQR 96.7–127.3 [11–13.8%]; Asia vs Middle East *p*=0.001; Asia vs Africa *p*=0.999; Middle East vs Africa *p*=0.107). Although patients from Africa had the highest HbA_1c_ value at diagnosis, this was not significantly different from other regions due to the low sample size. The percentage of patients treated with oral glucose-lowering drugs was significantly higher in Asia (76.0%) than in the Middle East (44.4%) (*p*<0.001), but no differences were observed either between Asia and Africa (52.2%, *p*=0.098) or between the Middle East and Africa (*p*=0.999). Significant differences in the percentage of patients treated with insulin were found between the Middle East and Asia (33.3 and 57.7%, respectively, *p*=0.014) and between the Middle East and Africa (79.3%, *p*=0.004), but no significant difference was found between Asia and Africa (*p*=0.338).

## Discussion

Here we report an increase in the proportion of new diagnoses of type 2 diabetes in the population aged under 21 years documented in the SWEET Registry, which includes countries from all continents, over a 10-year period. Except for AS/AF, obesity was a feature that was present in more than 75% of children and young people newly diagnosed with Type 2 diabetes in all geographical areas analysed.

According to the ISPAD guidelines, if HbA_1c_ levels are lower than 69 mmol/mol (8.5%), the initial treatment should be metformin. If the HbA_1c_ is ≥69 mmol/mol (8.5%), the preferred treatment is insulin. In the latter situation, the patient is gradually shifted to metformin a few weeks after the diagnosis of type 2 diabetes is confirmed and when the glycaemic values return to a controlled state. The increasing proportion of patients treated with insulin in the first year of diagnosis suggests the difficulty of achieving good glycaemic control using only oral hypoglycaemic drugs and lifestyle interventions.

Our study showed differences in HbA_1c_ at diagnosis between sexes. These differences align with a recent report on a large cohort of patients at the onset of type 2 diabetes in the UK [9]. In that study, in younger age groups, the HbA_1c_ levels were higher in male participants than in female participants. However, after the age of 50 years, no differences between the sexes were observed. Further studies are needed to understand why male participants have higher HbA_1c_ levels at a younger age.

At diagnosis, Asian/African children and young people had the highest HbA_1c_ levels, the lowest BMI and the highest proportion of insulin treatment among all children and young people from all geographical areas. It is well documented that type 2 diabetes develops at a lower BMI in Asian people [10–12]. However, no further conclusions can be drawn due to lack of information on the sociodemographic characteristics of patients and their families. As obesity in AS/AF was much lower than in other areas, the question remains whether there have been delays in diagnosis in those areas due to lack of parental awareness of the symptoms and early signs of diabetes other than obesity. However, another possibility remains: is it possible that African children with type 2 diabetes have a phenotype that is different from that in other areas of the world?

Most new diagnoses of type 2 diabetes occurred after the age of 10 years, although 8% of patients were diagnosed at younger ages. A peak of newly diagnosed cases was observed between 13 and 15 years old (Fig. 2). It may be hypothesised that the decrease after the age of 15 years is due to patients being diagnosed in adult diabetes centres and thus not captured through this registry. The increasing proportion of cases during the early stages of puberty is consistent with the well-known phenomenon of pubertal reduction in insulin sensitivity that has been reported for both sexes and across ethnic groups [13, 14].

The BMI at diagnosis of type 2 diabetes was variable, with lower values in AS/AF (Fig. 5), but the median BMI-SDS was >2.5 at diagnosis in each two-year time block between 2012 and 2021 (Fig. 4). Furthermore, it is interesting to note that 75% of the patients had BMI values above the 97th percentile at the time of diabetes diagnosis. In order to prevent type 2 diabetes, ISPAD guidelines recommend targeted screening after the onset of puberty or after 10 years old in young people with a BMI equal to or greater than the 85th percentile for their age and sex and risk factors for type 2 diabetes [6]. Our findings highlight the need for greater efforts to prevent, detect and treat weight excess before puberty, as a preventive measure for youth-onset type 2 diabetes. In addition, our results showed that, by waiting until a child reaches 10 years old for type 2 diabetes screening, at least 8% of patients may go undetected.

These results are consistent with those of other studies focusing on the increasing prevalence and incidence of type 2 diabetes in young people, as recently reported in a narrative review [4]. The increase in the proportion of new cases of type 2 diabetes during the COVID-19 pandemic should not lead to the assumption that there has been a reduction in the numbers of new cases of type 1 diabetes. Several studies have examined the impact of the COVID-19 pandemic on the incidence of both type 1 and type 2 diabetes [15–17]. A recent meta-analysis that included 17 studies of 38,149 young people [15] indicated an increase in the incidence rates of type 1 diabetes among children and young people after the onset of the pandemic, compared with the period before the pandemic. However, conclusive results are not yet available for type 2 diabetes. Furthermore, it is necessary to remember that the design of this study does not allow us to correlate the frequency of type 1 and type 2 diabetes, which instead requires population studies. A large cohort study in the USA reported an increase in the incidence of type 2 diabetes after the pandemic, from 14.8 per 100,000 person-years in 2016–2019 to 24.7 per 100,000 person-years in 2020 [18]. Therefore, the effects of the COVID-19 pandemic on diabetes prevalence are not conclusive, and further population-based studies are needed to provide more evidence.

This study has some limitations, including a lack of information on ethnic minorities, social and cultural environment, lifestyle, family history of diabetes, comorbidities and complications at diagnosis. Additionally, centres participating in the SWEET Registry may change from year to year or may not have been able to collect data over a certain period (e.g. during the COVID-19 pandemic), so there may be large proportions of missing data for some variables of interest. Consequently, due to limitations in the number of patients and the completeness of variables, especially in regions with lower coverage, such as Africa and Asia, the study may be affected by potential biases that reduce the generalisability of the results. Furthermore, as there are still few Asian and African centres participating in the SWEET Registry, there may have been insufficient data to draw strong conclusions when in fact differences exist. In fact, a recent study reported a more than 5.5-fold increase in the prevalence of type 2 diabetes from 2002 to 2016 among Korean children and adolescents aged 5 to 19 years [19]. Another limitation is that not all centres participating in the SWEET Registry documented the antibody status of patients classified as having type 2 diabetes, and we relied on provider diagnosis for diabetes types. Therefore, it has not been possible to use autoantibody measurements to eliminate potential type 1 diabetes cases that were incorrectly classified as type 2 diagnoses. In addition, with regard to completeness of the data, it is plausible that some patients are lost due to the transition from paediatric to adult centres for diabetes, and as a result the SWEET Registry may not have accurately captured the proportion of type 2 diabetes in patients who are 16–21 years old.

The results of this study show large geographical differences in the percentage of patients treated with insulin and oral agents at diagnosis. The proportion of children and adolescents treated with insulin increased from less than 10% in 2012–2013 to more than 30% in 2020–2021. The use of oral agents appears to be relatively stable over time (between 40% and 60%). Further studies are needed to establish whether these differences depend on therapeutic choices based on lifestyle, nutrition or physical activity rather than access to pharmacological treatments or whether they are due to different subtypes or severity of diabetes. Furthermore, although the use of injectable drugs was low during the study period, an increase may be expected in the near future, especially in high-income countries where glucagon-like peptide receptor agonists and dual incretin therapies may become accessible and publicly funded.

In conclusion, the SWEET Registry, an important worldwide tool to monitor the development and distribution of diabetes types, showed an increase in the proportion of new cases of type 2 diabetes in the decade 2012–2021. The increasing incidence of young people with type 2 diabetes in all continents demonstrates the urgent need for improved prevention interventions across the globe.

## Supplementary Information

Below is the link to the electronic supplementary material.

ESM (PDF 110 KB)

## Data Availability

Access to the programming code may be provided by the corresponding author if requested. For reasons of data protection, data at the individual level cannot be provided.

## Abbreviations

AS/AF: Asia/Middle East and Africa
AU/NZ: Australia and New Zealand
BMI-SDS: BMI SD score
EU: Europe
ISPAD: International Society for Paediatric and Adolescent Diabetes
NA: North America
SA: South America
