# Supplementary material for: Frequency and clinical characteristics of children and young people with type 2 diabetes at diagnosis from five world regions between 2012 and 2021: data from the SWEET Registry
Source: Diabetologia. 2024 Nov 6;68(1):82–93. doi: 10.1007/s00125-024-06283-5 (PMC11663186; doi:10.1007/s00125-024-06283-5)
Supplement: Supplementary file 1 — ESM (PDF 110 KB) [file 125_2024_6283_MOESM1_ESM.pdf]

Members of the SWEET Study Group

USA, Cincinnati: Cincinnati Children's Hospital
Bangladesh, Dhaka: BIRDEM, Diabetic Association of Bangladesh
USA, Stanford: Lucile Packard Children’s Hospital
USA, Denver Colorado: Barbara Davis Center
Australia, Perth: Princess Margaret Hospital for Children
USA, Boston: Boston Children’s Hospital
New Zealand, Auckland: Auckland Starship Hospital
UK, London: Barts and the London NHS Trust
UK, Birmingham: Birmingham Children’s Hospital
Turkey, Duzce: University of Duzce, Department of Pediatric Endocrinology
Korea, Seongnam: Seoul National University Bundang Hospital
Austria, Vienna: Universitätskinderklinik Wien
UK, Leeds: The Leeds Paediatric Diabetes Service at the Leeds Children’s Hospital
Germany, Hannover: Kinderkrankenhaus Auf der Bult
Canada, Calgary: Alberta Health Services
Costa Rica, San Jose: National Children’s Hospital, Hospital CIMA
Denmark, Herlev: Herlev University Hospital
Canada, Markham: Markham Stouffville Hospital
Italy, Florence: Meyer Children’s Hospital
Netherlands, Rotterdam: Diabeter Nederland
Argentina, Buenos Aires: Hospital Juan P. Garrahan
Italy, Mailand: Ospedale San Raffaele
Canada, Halifax: IWK Health Centre
Mali, Bamako: NGO Santé Diabète/Hopital du Mali
Greece, Thessaloniki: Hippokration Hospital of Thessaloniki
Hungary, Budapest: Semmelweis University
Sweden, Gothenburg: The Queen Silvia Children’s Hospital
Luxembourg, Centre Hospitalier de Luxembourg
Japan, Osaka: Department of Pediatrics Osaka City University Graduate School
Italy, Turin: Centro Diabetologia Pediatrica
Romania, Buzias: Clinical Center Cristian Serban
France, Paris: Hopital Necker Enfants Malades
Slovenia, Ljubljana: University Children’s Hospital
Italy, Ancona: Salesi University Hospital
Spain, Barcelona: Hospital Sant Joan de Deu
Canada, Vancouver: British Columbia Children’s Hospital
Germany, Leverkusen: Klinikum Leverkusen – Kinderklinik
France, Bordaux: Centre Hospitalier Universitaire de Bordeaux
Maldives, Male: Diabetes Society of Maldives
New Zealand, Christchurch: University of Otago and Canterbury District Health Board
Portugal, Lisbon, Estafania: Hospital Dona Estefania
Belgium, Leuven: University Hospital Leuven (UZ Leuven)
Canada, Sherbrooke: Sherbrooke University
Poland, Warsaw: Medical University of Warsaw
Ireland, Cork: Cork University Hospital

Lithuania, Kaunas: Hospital of LUHS Kauno Klinikos
Morocco, Rabat: Children’s Hospital – Unit Of Pediatric Diabetology
UK, Mansfield: Sherwood Forest Hospital
Australia, Newcastle: John Hunter Children’s Hospital
Italy, Verona: Universita di Verona
Turkey, Ege University Faculty of Medicine
Denmark, Aarhus: University of Aarhus
Croatia, Zagreb: University Clinical Hospital Center Sestre Milosrdnice
Sweden, Uddevalla: Uddevalla Children’s Hospital
India, Kota Rajasthan: Ramchandani Diabetes Care and Research Centre
Portugal, Lisbon: APDP-Portuguese Diabetes Association
Greece, Athens: P&A Kyriakou Children’s Hospital
Croatia, Zagreb: University Hospital Zagreb
Bulgaria, Sofia: University Paediatric Hospital
Czech Republic, Prague: University Hospital Motol Prague
Bulgaria, Varna: University Hospital St. Marina
Romania, Bucharest: Diabetes Nutrition and Metabolic Diseases Clinic DiabNutriMed
India, Chennai: MV Diabetes center
Norway, Haugesund: Helse Fonna
Greece, Thessaloniki: AHEPA University Hospital, 2nd Department of Paediatrics, Aristotle University of Thessaloniki
India, Mumbai: Conquer Diabetes
India, Ahmedabad: Diacare Clinic
Ireland, Dublin: Our Lady’s Children’s Hospital
Israel, Petah: Schneider Children’s Medical Center of Israel, Endocrinology
India, Ahemedabad: Swasthya Diabetes Care
Italy, Rom: Bambino Gesu Children’s Hospital
India, Sangli: Grow Kids Endocrine Clinic Sangli
Poland, Rzeszow: University of Rzeszow, Pediatric Endocrinology and Diabetes
Ireland, Limerick: University of Limerick
Czech Republic, Prague: Charles University Prague
Portugal, Porto: Centro Hospitalae S. Joao
Mauritius, Vacoas: T1Diams
Serbia, Belgrade: Institute for Mother and Child Healthcare
Poland, Katowicze: Medical University of Silesia
India, Nagpur: Diabetes Care & Research Centre
India, Coimbatore: PSG Institute of Medical Sciences
Chile, San Felipe: Hospital San Camilo
Spain, Barakaldo: Hospital Universitario Cruces
Haiti, Port-Au-Prince: Fondacion Hatienne de Diabete
